# Lidocaine and Pain Management in the Emergency Department: A Review Article

**DOI:** 10.5812/aapm.15444

**Published:** 2014-02-15

**Authors:** Samad EJ Golzari, Hassan Soleimanpour, Ata Mahmoodpoor, Saeid Safari, Alireza Ala

**Affiliations:** 1 Medical Philosophy and History Research Center, Tabriz University of Medical Sciences, Tabriz, Iran; 2 Cardiovascular Research Center, Tabriz University of Medical Sciences, Tabriz, Iran; 3 Department of Anesthesiology and Critical Care Medicine, Tabriz University of Medical Sciences, Tabriz, Iran; 4 Department of Anesthesiology and Critical Care, Iran University of Medical Sciences, Tehran, Iran; 5 Department of Emergency Medicine, Tabriz University of Medical Sciences, Tabriz, Iran

**Keywords:** Lidocaine, Pain Management, Emergency Department

## Abstract

**Context:**

In the present review, the analgesic effects of lidocaine in acute or chronic painful conditions in the emergency department are discussed. Lidocaine, as a medium-acting local anesthetic with short onset time, is well-recognized, not only as a valuable medication for numerous neuropathic pain conditions, but also for the management of both acute and chronic pain.

**Evidence Acquisition:**

Research studies related to the different applications of lidocaine in the emergency department were collected from different databases including Cochrane library, Medline (Ovid) and PubMed. The pooled data were categorized, summarized and finally compared.

**Results:**

Our study revealed that lidocaine is broadly used in various therapeutic approaches for different types of pain, such as visceral/central pain, renal colic etc., in the emergency department.

**Conclusions:**

The antinociceptive properties of lidocaine are derived from multifaceted mechanisms, turning it into a medication that is safe to administer via different routes which makes it available for use in a variety of medical conditions.

## 1. Context

Local anesthetics halt impulse initiation and transmission process in the axons, by blocking voltage-dependent sodium channels. Common local anesthetics are categorized into two major chemical classes: amino esters and amino amides. Amino esters are metabolized by plasma esterases, while amino amides are metabolized in the liver by hepatic enzymes (1).

Lidocaine, the most-frequently applied local anesthetic of the amide group, is used broadly in different fields of medicine; e.g. antiarrhythmic therapy, in addition to its administration as a local anesthetic (Table 1) (2-25).

**Table 1.** Lidocaine Applications in Different Studies

Clinical Application	Route of Administration	Studies	Year
**Renal Colic**	Intravenous	Soleimanpour et al. (1)	2012
	Intravenous	Soleimanpour et al. (5)	2011
	Local infiltration	Iguchi et al. (6)	2002
	Nerve block	Nikiforov et al. (7)	2001
**Antidysrhythmic**	Intravenous	Desouza et al. (3)	2013
**Wound**	Topical	Du et al. (4)	2012
**Neuropathic Pain**	Topical	Sawynok et al. (8)	2013
	Subcutaneous	Brose et al. (9)	1991
	Subcutaneous	Schwager et al. (10)	1995
	Intravenous	Tremont-Lukats IW et al. (11)	2005
**Biopsy Sampling**	Topical	Olad-Saheb-Madarek et al. (12)	2013
**Post**-**Stroke Pain**	Intravenous	Edmondson et al. (13)	1993
**CRPS ** ^**a**^	Subcutaneous	Linchitz et al. (14)	1999
	Intravenous	Monacelli et al. (15)	2012
	Intravenous	Kosharskyy et al. (16)	2013
**Post-herpetic Neuralgia**	Intravenous	Baranowski et al. (17)	1999
**Post**-**Amputation**	Intravenous	Wu et al. (18)	2002
**Headache**	Intravenous	Hand et al. (19)	2000
	Intravenous	Krusz et al. (20)	2006
	Intravenous	Rosen et al. (21)	2009
**Terminally-Ill Pain**	Intravenous	Ferrini et al. (22)	2000
**Hematoma Block**	hematoma block	Dorf et al. (23)	2006
**Intra-Articular**	Intra-articular	Waterbrook et al. (24)	2011
		Brickman et al. (2)	2013
**Nerve block**	Nerve block	Wedel et al. (25)	2007
		Nikiforov et al. (7)	2001

^a^ Abbreviation: CRPS, complex regional pain syndrome.

The analgesic properties of intravenous (IV) lidocaine were first reported in cancer and postoperative patients. Later, lidocaine was shown to provide analgesia, by blocking both peripheral and central voltage-dependent sodium channels. In the IV administration route, it can also relieve both deafferentation and central pain. The antinociceptive properties of lidocaine seem to be derived from a more multifaceted process, rather than simple inhibition of neuronal ectopic discharges (26).

While lidocaine is efficient for both visceral and central pain, its IV form can be considered as a proper choice in scenarios where opioids are either ineffective or associated with undesirable complications. In addition to its extensive applications, lidocaine can be administrated via various routes (i.e. IV, subcutaneously (SC) and nerve blocks). Intravenous lidocaine is used broadly in the management of neuropathic pain, postoperative pain, postherpetic neuralgia, centrally mediated pain, headache and infiltrative malignant neurological lesions (8). Lidocaine is a relatively safe drug, which can be used at low doses without any notable safety concerns. Sensitivity to lidocaine, a hazardous yet extremely rare complication, might be associated with dyspnea and increased incidence of cardiac dysrhythmia. The most frequently reported complications, including periorbital numbness, dizziness, vertigo and dysarthria, are due to lidocaine accumulation in the body (8, 26). Less frequent side effects, such as tachycardia, allergic reactions, dry mouth, insomnia, tremor, and metallic taste, are occasionally reported. Lidocaine is inexpensive and easy to access. Complications are much less encountered when using lidocaine compared with opioids and other analgesics (27). Interestingly, side effects of IV lidocaine are predictable, providing a broad safety margin. Due to the short half-life of lidocaine, its toxicity symptoms are transient and rapidly reversible, adding to its popularity amongst physicians working in the emergency departments and hospitals (8). As previously described, lidocaine has a wide range of applications in the management of neuropathic pain, postoperative pain, postherpetic neuralgia, centrally mediated pain, headache and infiltrative malignant neurological lesions (1, 8).

## 2. Evidence Acquisition

Scientific search engines including Cochrane library, Medline (Ovid) and PubMed were used to collect different types of articles, including systematic reviews, evidence-based clinical practice guidelines, health technology assessments, and randomized controlled trials related to lidocaine and its administration in the emergency department. The following key words were used: Lidocaine; Therapeutic approaches; Treatment; Emergency department.

Our search resulted in 548 articles. The irrelevant papers by title review (332) were excluded, leaving 216 articles. Twenty-nine articles were selected for further use; the remaining 187 articles were excluded due to the following reasons: irrelevant abstracts or full-text review, duplicate records and lack of access to the full-text of the articles.

## 3. Results

In the following sections, indications forlidocaine administration for pain management in the emergency department is discussed:

### 3.1. Visceral/Central Pain

Intravenous lidocaine infusion has been suggested to be effective in the management of both visceral and central pain, even in patients in whom opiates cause side effects and fail to provide appropriate analgesia (8-10, 12-14, 17-19).

### 3.2. Renal Colic

#### 3.2.1. Trigger Point Injection Approach

This approach has been successfully used in the management of renal colic. In a study, patients who received a local injection of lidocaine 1% (10-15 mL) in the renal colic trigger point, were reported to experience significantly less pain, compared to their counterparts who had received an IV injection of analgesic combination [butylscopolamine bromide (40 mg), Sulpyrine (500 mg) and glucose 5% solution (20 mL)]. Success rates of 29/30 versus 22/30 were reported in the lidocaine versus butylscopolamine groups, respectively (i.e. only one patient in the lidocaine group required supplementary analgesia). No complications were reported in the lidocaine group. Hence, trigger point injection approach using lidocaine is a safe, easy and efficient method in the management of renal colic (6).

#### 3.2.2. Intravenous Approach

Lidocaine alters sympathetic smooth muscle tone by reducing the transmission in the afferent sensory pathways. Ultimately, considerable pain reduction could be achieved by administration of IV lidocaine, a possibility that has turned lidocaine into a suitable alternative for cases in which opioids are ineffective or associated with undesirable complications (1).

In a study performed on eight patients with renal colic refractory to nonsteroidal anti-inflammatory drugs (NSAIDs) and opioids, IV infusion of lidocaine (1.5 mg/kg in 5 minutes) was used. Interestingly, the mean visual analogue scale (VAS) scores decreased from 8.87 to 1, in just 30 minutes after receiving the IV analgesic. Colic pain disappeared completely within 10, 20, and 30 minutes in four, 68, and 78 patients, respectively. Regarding the complications, two and three patients experienced transient mild dizziness and minimal slurring of speech, respectively, while no serious adverse event was reported. Complete pain relief was reported in seven patients after lidocaine administration, which lasted until hospital discharge. One patient required supplementary analgesia after 30 minutes. In the follow-up period, renal colic recurrence was reported in six patients, which was managed by NSAID therapy (5).

In another study performed on 240 renal colic patients referred to an emergency department, the efficacy of intravenous morphine versus lidocaine was compared. Patients in the lidocaine group received IV lidocaine 2% (1.5 mg/kg) and those in morphine group received IV morphine solution (0.1 mg/kg). Success in pain management was defined as pain score of less than 3 for 30 minutes after the last analgesic dose, or if all the 10mL of solution in the syringe was used up. Proper response to treatment was observed in 90% vs. 70% of the patients in the lidocaine vs. morphine groups (P = 0.0001). No serious or life-threatening complication was reported in any of the lidocaine group patients, emphasizing the fact that IV lidocaine is a safe and efficient choice in patients with renal colic (1).

#### 3.2.3. Subcutaneous Paravertebral Block Approach

Subcutaneous paravertebral block was first used successfully in the treatment of renal colic in a pregnant woman with gestational age of 29 weeks, who was referred to an emergency center with severe pain in the right flank. Administration of meperidine (150 mg) and morphine (8 mg) failed to reduce her severe pain. Subcutaneous paravertebral block with lidocaine 2% (6 mL) was considered and performed in left lateral decubitus position. Patient’s pain decreased dramatically from 10/10 to 2/10 and 0/10 at 5 and 10 minutes after the block, respectively. However, her pain increased to 7/10 just 2 hours later. Consequently, she was given a SC injection of bupivacaine 0.25% (6 mL), which was effective and the patient was pain-free for another 2 hours. Subsequently, analgesia was maintained by IV meperidine (75 mg). Afterwards, spinal anesthesia was performed for the patient to undergo nephrostomy and pelvic stenting (7).

### 3.3. Terminally Ill Patients

Intravenous lidocaine infusion has been reported to be effective as a last resort for the refractory pain in terminally ill patients. In a 51 year old female patient with primary neuroectodermal tumor and severe pain (10/10) due to spinal compression at T6-T8, IV morphine (bolus dose of 25 mg and infusion rate of 50 mg/h) was reported to have failed to reduce pain. Other supplementary analgesics were tried, which were of no significant efficacy. Hence, IV lidocaine was considered with an initial treatment course consisting of 100 mg lidocaine (1.5 mg/kg) within 20 minutes, which associated with a dramatic decline in the pain score of the patient. Consequently, IV lidocaine infusion at the rate of 100 mg/h was selected for analgesia maintenance. Throughout treatment course, different routesof lidocaine administration were examined; bolus and continuous infusion of lidocaine were the most effective approaches (22).

### 3.4. Headache

Although lidocaine is not recognized as a first-line choice in the treatment of migraines, it might be considered as a part of the daily therapeutic regimen in order to alleviate uncontrollable headaches. The hypothesis is to saturate the Na^+^ channels very slowly in order to achieve the most appropriate blockade. The main purpose of such a regimen is to allow time for other treatments to take effect, as the lidocaine treatment course is often not long-lasting (lasting for less than 48 hours). Intravenous lidocaine and calcium channel blockade (through IV MgSO_4_) can be particularly effective along with IV dexamethasone, especially for chronic daily headache (CDH).However, much of its therapeutic mechanism remains to be discovered (20).

In a study performed on CDH patients, IV lidocaine infusion succeeded in reducing the mean VAS of the patients from 7.9 to 3.9, within 8.5 days (21). In another study focusing on the intravenous treatment of the chronic headache in patients referred to out-patient clinics, it was concluded that the response to IV lidocaine was far better for patients with CDH (20).

### 3.5. Postherpetic Neuralgia

Lidocaine infusion has been suggested to be efficient in postherpetic neuralgia (PHN), by several studies. In one of them, the effect of IV lidocaine at two doses (0.5 mg/kg/h and 2.5 mg/kg/h over 2 hours) on PHN pain and allodynia was evaluated. A significant effect on PHN pain and allodynia following a brief IV infusion of lidocaine was observed, indicating the fact that lidocaine infusion might be effective in PHN (17).

### 3.6. Post-Stroke Pain Syndrome

Considered to be one of the most refractory pain challenges, post-stroke pain syndrome (PSPS) seems difficult to treat. In a study performed on four patients with refractory PSPS, 48-hours infusion of lidocaine was administered after an initial intravenous bolus of 50-100 mg. Pain scores of all patients decreased significantly within the first 12 hours of infusion; therefore, to maintain the analgesia, mexiletine (an oral congener of lidocaine) was administered. Later, 50% of the patients pursued taking the medication with an outstanding pain relief throughout the 12 month follow-up period, while 50% of the patients failed to continue the treatment course due to the emergence of side effects. Consequently, an algorithm was proposed for the treatment of PSPS (16).

### 3.7. Complex Regional Pain Syndrome

Previously known as reflex sympathetic dystrophy (RSD) and causalgia, complex regional pain syndrome (CRPS) types I and/or II are challenging medical conditions which can be associated with other symptoms such as allodynia, hyperpathia, dysesthesia, changes in hair and nail growth, decreased range of motion of the involved extremities and color and temperature changes. In a study performed on CRPS patients, continuous 4 - 8 weeks subcutaneous infusion of lidocaine 10% was considered. The regimen significantly decreased the pain scores in most of the patients, an effect that persisted after termination of the continuous subcutaneous infusion as well. However, in some cases, periodic maintenance infusions was required (15).

### 3.8. Neuropathic Pain

Neuropathic pain is defined by the International Association for the Study of Pain as “pain resulting from damage to the peripheral or central nervous system”. It is characterized by major clinical features such as lancination and spontaneous character, variable onset after injury, association with allodynia and hyperesthesia, and evoked summation and hyperpathia. Densely expressed sodium channels in injured peripheral nerves are blamed for creating persistent spontaneous discharges, resulting in a central hyperexcitable state. Lidocaine inhibits ectopic discharges originating from the injured nerve, the dorsal root ganglion, and peripheral neuromata (11). Subcutaneous infusion of lidocaine 10% has been shown to be effective in the treatment of neuropathic pain (9).

### 3.9. Intra-Articular Lidocaine Injection for Shoulder Reductions

Shoulder dislocations are common and many of them are reduced without any or proper analgesia, in either outpatient or emergency settings. The use of intra-articular lidocaine injection for reduction of anterior shoulder dislocations has been reported to be helpful in both outpatient clinical settings and emergency departments (24).

### 3.10. Topical Anesthetics

Topical lidocaine is available in solution, gel, and ointment forms. Viscous Lidocaine can be utilized temporarily on the inflamed mucous membranes. For catheter insertion (Foley catheters and nasogastric tubes) or similar painful procedures, lidocaine gel is an excellent choice. However, special attention must be devoted not to exceed maximal doses while using any form of the medication. Diverse combinations of topical anesthetics mostly contain lidocaine, such as lidocaine, epinephrine and tetracaine (LET), or lidocaine and prilocaine (EMLA). The LET combination is commonly used in infiltrative anesthesia and has the advantages of maintaining proper homeostasis, painless administration, no injection requirement, and not distorting wound edges. In order to achieve the maximal effect, LET should be applied for at least 20 minutes and it should not be used on mucous membranes or in end-artery fields. The EMLA cream is a 1:1 combination of lidocaine 2.5% and Prilocaine 2.5%, which is used preoperatively in pediatric patients. It is recommended to be applied for 45 minutes to 2 hours, and it provides abundant analgesia for minor procedures, to a satisfactory degree of pain tolerance (28).

### 3.11. Intravenous Regional Anesthesia: Bier Block

First introduced by August Bier in 1908, this block aims at exsanguinating the extremity by an arterial tourniquet. Subsequently, local anesthetic is injected into the venous system of the extremity in order to provide anesthesia. The block is mostly used in surgical procedures that will be completed within 40-60 minutes and involve the forearm and leg (open procedures or closed reductions). Numerous mechanisms have been proposed for intravenous regional anesthesia, yet the exact mechanism remains to be discovered. Early anesthetic action of the local anesthetic is due to its effect on major nerve trunks, small nerves, and nerve endings. Associated hypothermia and acidosis are considered as local anesthetic activity enhancing factors. Lidocaine 0.5% (maximum dose of 3 mg/kg) is considered an excellent choice as it has a relatively low toxicity and high therapeutic index. Lidocaine should not contain epinephrine or any preservatives as it would lead to catastrophic consequences (25, 29).

### 3.12. Local and Regional Anesthesia

Lidocaine, with an action onset of 2-5 minutes and duration of 1-2 hours, is an unsurpassed choice. Pain on injection site caused by lidocaine could be diminished by adding bicarbonate (in a proportion of 1 NaHCO3:9 lidocaine), using needles with small gauge (27 or 30 gauge), warming the solution prior to injection and the last, but not the least, slow injection of the solution. Epinephrine, if added to lidocaine, could enhance the blocking and provide longer durations of anesthesia, wound homeostasis, less systemic absorption and, consequently, decreased toxicity. It, however, would lower the pH of the solution and increase the pain on the injection site. Based on the fact that epinephrine reduces local perfusion, it should not be used in the ring block of the digits, penis, nose, ears or where there is risk of ischemia. The maximum dose of lidocaine is 4.5 mg/kg without epinephrine, and 7 mg/kg with epinephrine. However, the maximum safe dose in intercostal nerve block is 10 times less than the standard doses. In order to avoid any allergic reactions in patients with history of allergy to any local anesthetic, skin test with 0.1 mL of preservative-free anesthetic from another class of local anesthetics should be performed. Alternatively, diphenhydramine (0.5 to 1%) can be used simultaneously (1, 22, 30).

### 3.13. Local Anesthetic Infiltration

Local anesthetic (LA) infiltration is the most common use of LAs, which can provide anesthesia for numerous invasive procedures. Wound or incision margins are infiltrated by LA, or LA is injected directly into the wound, which is called “field block”. In order to avoid any further pain in an intact skin, raising a skin wheal would prevent further pain caused by consequent infiltration. The LA can also be used in orthopedic procedures, such as fracture and joint reduction, by directly injecting LA into the affected joint or fracture hematoma (21, 30).

Axillary brachial plexus block, first described by Halstead in 1884, is an excellent choice for the procedures performed on the forearm and hand injuries and fractures. Reasonably large volumes of local anesthetic (35-40 mL) are required in order to guarantee complete anesthesia. Lidocaine 2% in combination with bicarbonate and epinephrine would provide analgesia for 3-6 hours, with an onset time of 5-15 minutes. In this block, slow approaches with frequent aspirations are recommended as the injection field is highly vascular and inadvertent intravascular injection is probable (30).

### 3.14. Digital Blocks

Digital blocks performed on fingers or toes are uniquely valuable, as fewer volumes of anesthetics are required. However, a better-quality anesthesia could be achieved. Nevertheless, delayed onset of action is inevitable. Prior to block, neurovascular examination must be performed and epinephrine administration should be avoided. Although rare, complications include nerve injury and systemic toxicity following intravascular injection (31).

### 3.15. Hematoma Blocks

Hematoma block with lidocaineis regularly used in the emergency departments for the reduction of Colles' fracture. Lidocaine 2% (5-10 mL) is directly injected into the fracture hematoma. Although hematoma block is an extremely practical approach, lidocaine toxicity is one of its few potential complications which could be prevented by proper dosing of the LA (23).

## 4. Conclusions

Lidocaine is a safe medication that is administered via different routes in different fields of the emergency medicine including visceral/central pain, renal colic, terminally ill patients, headache, postherpetic neuralgia, post-stroke pain syndrome, CRPS, neuropathic pain, intra-articular injection, topical anesthesia, Bier block, local and regional anesthesia, local anesthetic infiltration, digital blocks and hematoma blocks.
